# Cooling effect and control factors of common shrubs on the urban heat island effect in a southern city in China

**DOI:** 10.1038/s41598-020-74559-y

**Published:** 2020-10-14

**Authors:** Rongfei Zhang

**Affiliations:** 1 Guangdong Engineering Center of Non-Point Source Pollution Control, Guangdong Key Laboratory of Integrated Agro-Environmental Pollution Control and Management, Institute of Eco-Environmental and Soil Sciences, Guangdong Academy of Sciences, Guangzhou, 510650 People’s Republic of China; 2 National Regional Joint Engineering Research Center for Soil Pollution Control and Remediation in South China, Guangzhou, 510650 People’s Republic of China; 3 Guangdong Academy of Sciences, Guangzhou, 510070 Guangdong People’s Republic of China

**Keywords:** Plant sciences, Structural biology, Systems biology, Climate sciences, Environmental sciences

## Abstract

Because the heat island effect can make cities warmer than their surroundings, it can make urban dwellers uncomfortable and even affect their health, which is particularly pronounced in developed cities in southern China. To reduce the heat island effect and improve the environment, various types of vegetation have been planted in the urban green belt. Though previous studies have been conducted on the beauty, air purification functions and cooling effect of vegetation, little is concentrated on the different cooling effects and control factors of various common shrubs on the heat island effect in cities. In this study, five of the most regionally common shrubs were selected to study the cooling effect in Guangzhou, southern China. The maximum surface temperatures of five shrubs and pavement were compared using infrared temperature sensors from April 1st 2019 to October 31st 2019. Results show that (1) All five shrubs showed noticeable seasonal variation, and the average surface temperatures of the five shrubs were between 38.0 and 42.2 °C during May–August and 30.7–34.1 °C during the other seasons (April, September and October);. (2) *Murraya exotica* L. exhibited the best cooling effect on the maximum surface temperature. Its value was 44.7 °C, and the absolute difference values of *Murraya exotica* L. (10.3 ± 1.7 °C) were higher than any other shrub during the study period; (3) Both the LAI (*R*^2^ = 0.57, *p* < 0.01) and plant height (*R*^2^ = 0.13, *p* < 0.01) are control factors of the cooling effect on vegetation surface temperature for the five shrubs. This study revealed the differences in the cooling effect and influencing factors of five regionally common shrubs on the heat island effect. Research on the functional characteristics of plants and plant selection in urban green belts has both theoretical and practical significance.

## Introduction

An urban heat island (UHI) is an urban metropolitan area that is significantly warmer than its surrounding rural areas due to human activities and LUCC (land use and land cover) differences^1^. UHIs have many negative side effects. The UHI effect causes the highest temperatures in cities to exceed the breeding ranges of many animals and microbes^2^. UHIs may cause secondary effects on local meteorology, including changes in local wind, fog, relative humidity and rainfall^3^. They can also lead to greater upward motion, which may cause extreme weather events such as thunderstorms^4^. More importantly, UHIs affect the health and welfare of urban residents by increasing the magnitude and duration of heat waves in cities^5^.

Various studies have been conducted to better understand the impacts of UHIs^1,2,4,5^. For example, Salvati et al.^6^ studied the intensity of UHIs in Barcelona (Spain), the densest Mediterranean coastal city, and their impacts on the cooling demand of residential buildings. He found that during the daytime, air temperatures at the street level are higher than the roof level, and the energy impact of the UHI is more relevant for higher solar gains. Santamouris et al.^7^ studied the climate and energy potential of mitigation technologies in Sydney to decrease the energy impact of UHIs. Results showed that solutions involving the increase of the global albedo of the city demonstrate the highest benefits, achieving a reduction of peak ambient temperature of up to 3 °C. Singh et al.^8^ used Landsat thermal data and a field survey to study the negative impacts of urbanization, including rising temperatures and the degradation of urban ecology in Lucknow city, India. The results show that the spatial distribution of the land surface temperature was affected by the land use-land cover change and anthropogenic causes. Furthermore, the trend of global warming has not changed^9^. Ever-rising temperatures exacerbate the UHI effect in densely populated cities already filled with heat-amplifying surfaces like concrete, asphalt, and glass^6,7^. Therefore, it is an increasingly urgent task to reduce the greenhouse effect through multi-channels and multi-methods, such as green façade, green roof, green wall and urban green belts (park, grass land, trees and forests)^8,10,11^.

Urban green belts are used to beautify city landscapes and purify air, water and soil. They also have a mitigating effect on UHIs^12^. Various plants have been used to produce green belts,each plant (including trees, shrubs and grasses) performs a different ecological function^13–16^. Shrubs are widely used in green belts because of their small size, high plasticity, and diverse ecological and landscape functions, including complementing the space needed by herbs and trees^17–20^. A number of studies have been conducted on the effects and regulatory functions of shrubs on UHIs in different cities^21^. For example, Edmondson et al.^22^ studied the cooling effect of trees and shrubs on UHIs and found that they moderate soil surface temperature. Lin et al.^23^ compared the cooling effect of trees and shrubs on UHIs in high-rise, high-density environments. Cao et al.^24^ quantified the cooling effect of parks on UHIs by comparing the area of trees, shrubs and grasses inside parks in Nagoya, Japan. Tan et al.^25^ studied the impact of shrubs on temperature reduction through the evapotranspiration rate and albedo in the tropical outdoor environment. However, we are not aware of any studies that have compared the cooling effect and controls of different shrubs on UHIs.

The UHI effect is particularly pronounced in southern Chinese cities, such as Guangzhou, where are hot all the year round^26–29^. For hot and subtropical cities, a number of studies have been conducted to examine the UHI effect^30^. For example, Sun et al.^29^ studied the relationship between land surface temperature and LUCC in Guangzhou. Chen et al.^26^ used remote sensing models to simulate the UHI effect in Guangzhou. However, no studies have examined which vegetation type has the greatest cooling effect on UHIs in hot and subtropical cities, such as Guangzhou. Because the climatic, topographic and soil conditions of each city differ, it is necessary to conduct relevant studies in the city of interest for planning and vegetation selection. In addition, there are a variety of indicators to describe the reduction of urban greenhouse effect, such as the cooling effect of building protective materials on the rigid body, the reduction of heat absorption by exterior materials, and the reduction of ambient temperature^31^. In this study, as the object of study is the shrub, the temperature above the plant is taken as the descriptive indicator.

This study aims to (1) compare the diurnal maximum surface temperatures of pavement and shrubs in Guangzhou, China; (2) compare the cooling effect of different shrubs on surface temperature; (3) identify the factors that control the cooling effect of shrubs on surface temperature.

## Materials and methods

### Study area

**Figure 1:**
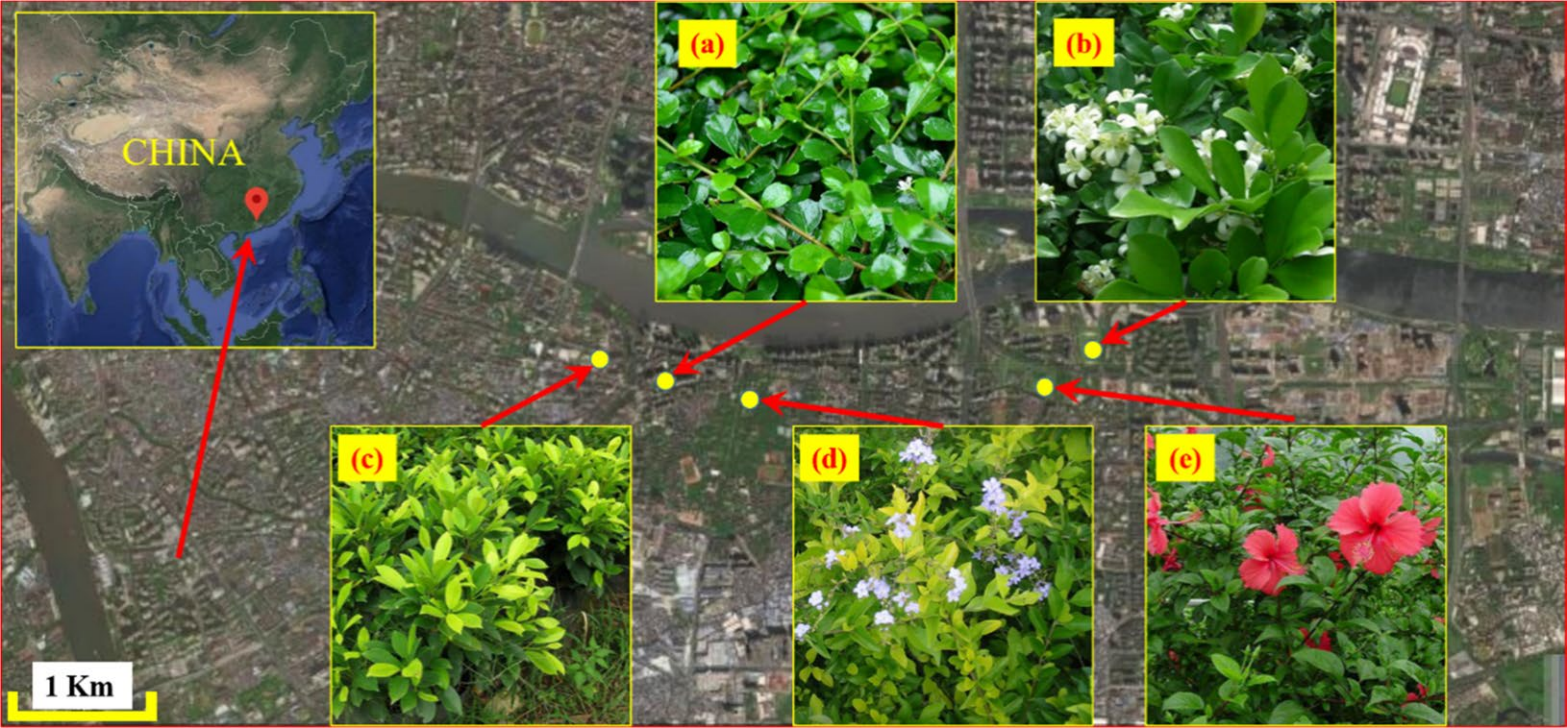
Study area and location of the study sites. Five regionally common shrubs were studied: *Carmona microphylla* (Lam.) G. Don (**a**), *Murraya exotica* L. (**b**), *Duranta repens* L. (**c**), Ficus microcarpa ‘Golden Leaves’ (**d**) and *Hibiscus rosa-sinensis* Linn (**e**).

The study area (22° 26′–23° 56′ N, 112° 57′–114° 3′ E) is located in Guangzhou of Guangdong province, southern China. Guangzhou is one of most developed cities in China, and is the core city of the Guang-Fo, Guangdong—Hong Kong—Macao and Pearl River Delta metropolitan area. Study sites (Fig. 1) were located in a green belt adjacent to concrete roads. Guangzhou is a hilly area with high terrain in the northeast and low terrain in the southwest, surrounded by mountains and sea. The north is hilly and mountainous with dense forests. The northeast is a middle-low mountain area. The middle is a hilly basin, and the south is the coastal alluvial plain that forms part of the Pearl River Delta. Guangzhou is a subtropical coastal city with a maritime subtropical monsoon climate. It is warm and rainy, with a long summer and short frost period. The annual average temperature is 20–22 °C. The hottest month of the year is July, with an average monthly temperature of 28.7 °C. The urban annual precipitation is 1720 mm.

### Field measurements

The species and number of the ten most regionally common shrubs are shown in Table 1 below. Five of these (*Carmona microphylla, Murraya exotica, Duranta repens, Hibiscus rosa-sinensis* and *Ficus microcarpus* cv.“Golden leaf”) were selected for study. The five shrubs were planted at the same time and all were grown in green belts on roadsides.

**Table 1:** Common shrub species for urban greening in Guangzhou.

Shrub types	Genus	Number (one)	Percent (%)
*Carmona microphylla*	*Borraginaceae*	476,020	21.1
*Murraya exotica*	*Rutaceae*	209,660	9.3
*Duranta repens*	*Verbenaceae*	187,194	8.3
*Hibiscus rosa-sinensis*	*Malvaceae*	141,924	5.9
*Ficus microcarpus* cv.“Golden leaf”	*Moraceae*	128,491	5.7
*Excoecaria cochinchinensis*	*Euphorbiaceae*	77,825	3.4
*Hamelia patens*	*Rubiaceae*	77,368	3.4
*Ligustrum sinense*	*Oleaceae*	71,325	3.2
*Bougainvillea glabra*	*Nyctaginaceae*	51,836	2.3
*Aglaia odorata*	*Meliaceae*	46,784	2.1

In this study, non-contact infrared temperature sensors (RS-WD-HW-I20, Shandong province of China) were used to measure surface temperature for pavement and shrubs. The temperature range for this kind of sensor is 0–1000 °C. For every shrub, the temperature sensor was tied to an upright stick so that the sensor could move as the shrub grew. All sensors were adjusted to the height of the plant to ensure that they were always above the plant, and they were always directly exposed to the sun. Temperature data were automatically and continuously monitored once every 5 min. The leaf area index (LAI) was measured once every 10–15 days using a LAI-2200C (LI-COR LTD., U.S.). The height of the five kinds of shrub was measured once every 10–15 days at the same time of day using a millimeter scale tape measure.

### Assessment indicators of the cooling effects of shrubs

In this study, the surface temperature of concrete pavement was taken as a reference point, then the maximum surface temperature and the temperature difference between the surface of concrete pavement and shrubs was used as an assessment indicator to evaluate the cooling effect. Humans are more sensitive to extreme heat, and the highest daily temperature poses the greatest threat to human health. Therefore, the maximum temperature is used as the assessment indicator. The greater the difference between the maximum surface temperature of the pavement and that of the shrubs, the better the cooling effect. The differences in surface temperature between shrubs and their average values were used to judge which had a greater cooling effect. The determination coefficient (*R*^2^) and linear relation formula were used to evaluate the relationship between the cooling effect and its controls, i.e. the correlation between monthly LAI (plant height) of shrubs and the difference in surface temperature between concrete pavement and plants.

## Results

### Difference in diurnal maximum surface temperature for pavement and shrubs

**Figure 2:**
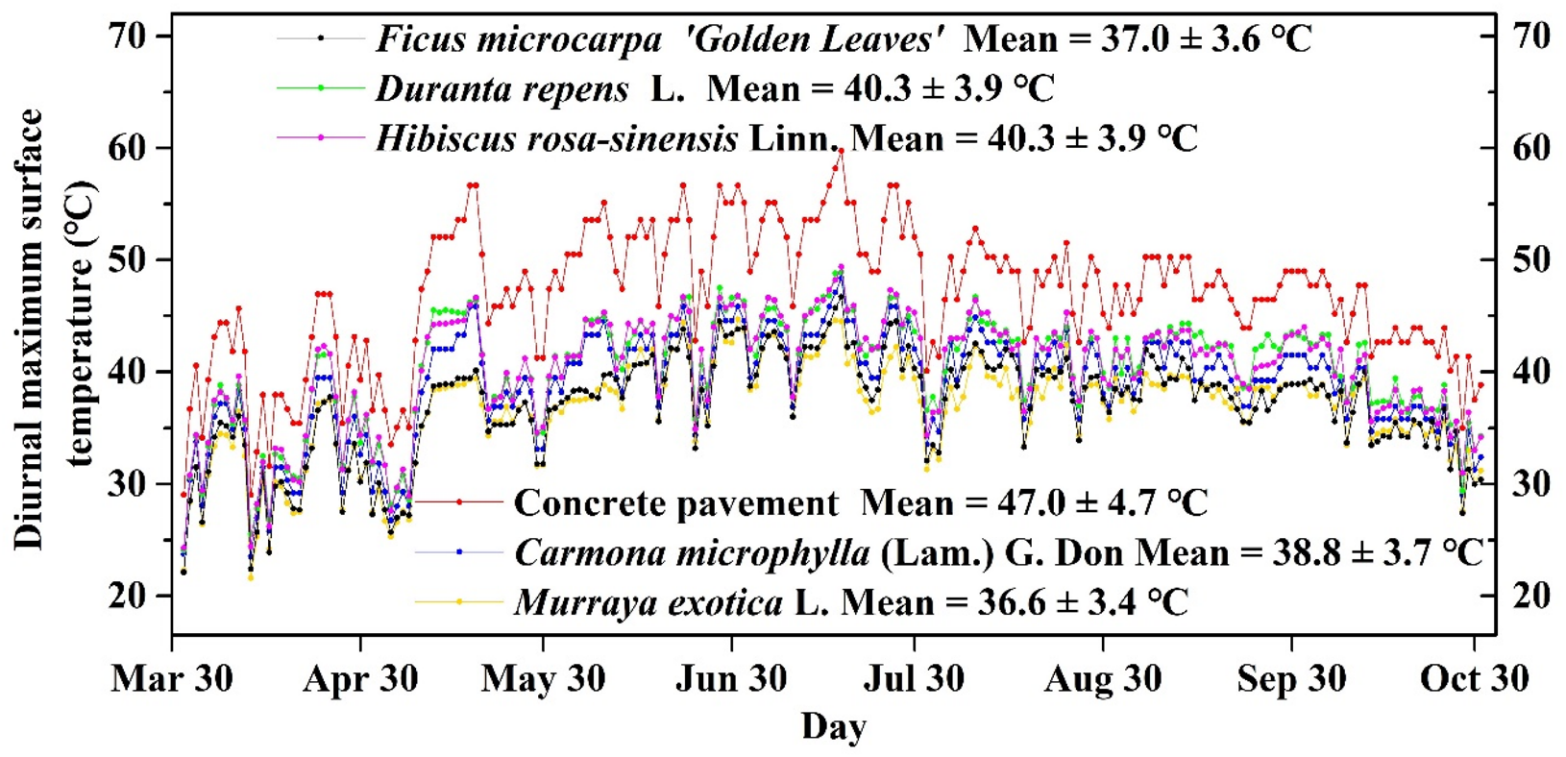
The highest daily air temperature on concrete pavement and five typical shrub types (*Carmona microphylla* (Lam.) G. Don, *Murraya exotica* L., *Duranta repens* L., *Ficus microcarpa* ‘Golden Leaves’ and *Hibiscus rosa-sinensis* Linn.).

As shown in Fig. 2, the diurnal maximum surface temperature exhibited similarities and differences for the five shrubs. Surface temperature was related to the surface type. Surface temperature for pavement was higher during May–August (Mean = 49.4 ± 4 °C) compared to other seasons (April, September and October: Mean = 39.3 ± 4 °C). All of the shrubs showed noticeable seasonal variation. Temperatures varied between 38.0 and 42.2 °C during May–August and 30.7–34.1 °C during the other seasons for the five shrub types.

Most importantly, the maximum diurnal surface temperature for pavement during the study period (April–October) was 59.7 °C. For the five shrubs, the highest value was 49.4 °C (*Hibiscus rosa-sinensis* Linn.). *Duranta repens* L., *Carmona microphylla* (Lam.) G. Don and *Ficus microcarpa ‘Golden Leaves’ were in the middle*. *Murraya exotica* L. exhibited the best cooling effect during the study period with a maximum surface temperature of 44.7 °C.

During the study period, the overall average diurnal maximum surface temperatures of pavement was 47.0 ± 4.7 °C. The average values of the five shrub types were, 40.3 ± 3.9 °C, 40.3 ± 3.9 °C, 38.8 ± 3.7 °C, 37.0 ± 4.7 °C and 36.6 ± 4.7 °C, for *Hibiscus rosa-sinensis* Linn, *Duranta repens* L., *Carmona microphylla* (Lam.) G. Don and *Ficus microcarpa 'Golden Leaves'* and *Murraya exotica* L., respectively.

### Cooling effect on the surface temperature above different shrubs

**Figure 3:**
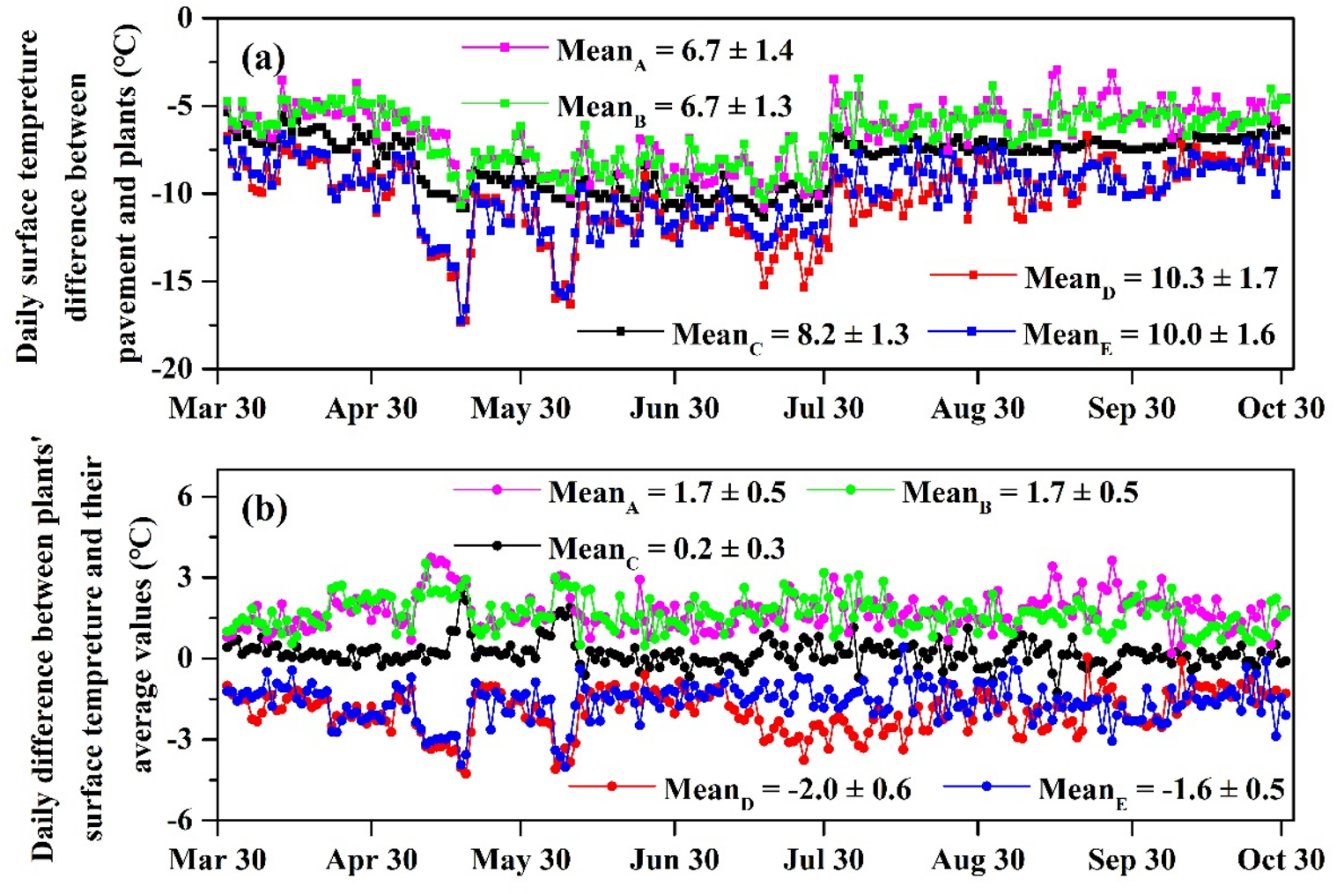
The daily temperature difference (**a**) between plant surface and pavement, Mean = Mean_shrub_ − Mean_pavement_ and (**b**) between plant surface and average values, Mean = Mean_shrub_ − Mean_average_. A, B, C, D and E represent *Duranta repens* L., *Hibiscus rosa-sinensis* Linn., *Carmona microphylla* (Lam.) G. Don, *Murraya exotica* L. and *Ficus microcarpa 'Golden Leaves',* respectively.

Figure 3a shows the difference values of diurnal maximum surface temperature between the pavement and the shrubs. The absolute difference values of *Murraya exotica* L. (10.3 ± 1.7 °C) and *Ficus microcarpa ‘Golden Leaves’* (10.0 ± 1.6 °C) exhibited higher values than *Duranta repens* L. (6.7 ± 1.4 °C), *Hibiscus rosa-sinensis* Linn (6.7 ± 1.3 °C) and *Carmona microphylla* (Lam.) G. Don (8.2 ± 1.3 °C). This means that *Murraya exotica* L. and *Ficus microcarpa 'Golden Leaves'* have a greater effect on cooling the surface temperature than the other three shrubs.

Figure 3b shows the difference values of diurnal maximum surface temperature between several shrubs and their average values. The difference values of *Duranta repens* L. (1.7 ± 0.5 °C) and *Hibiscus rosa-sinensis* Linn. (1.7 ± 0.5 °C) were noticeably positive and significantly higher than *Carmona microphylla* (Lam.) G. Don (0.2 ± 0.3 °C). This means that the cooling effect on the surface temperature of these three shrubs was below the average value. However, the cooling effect of *Murraya exotica* L. and *Ficus microcarpa ‘Golden Leaves’* is the same as the average value of the five shrubs.

**Figure 4:**
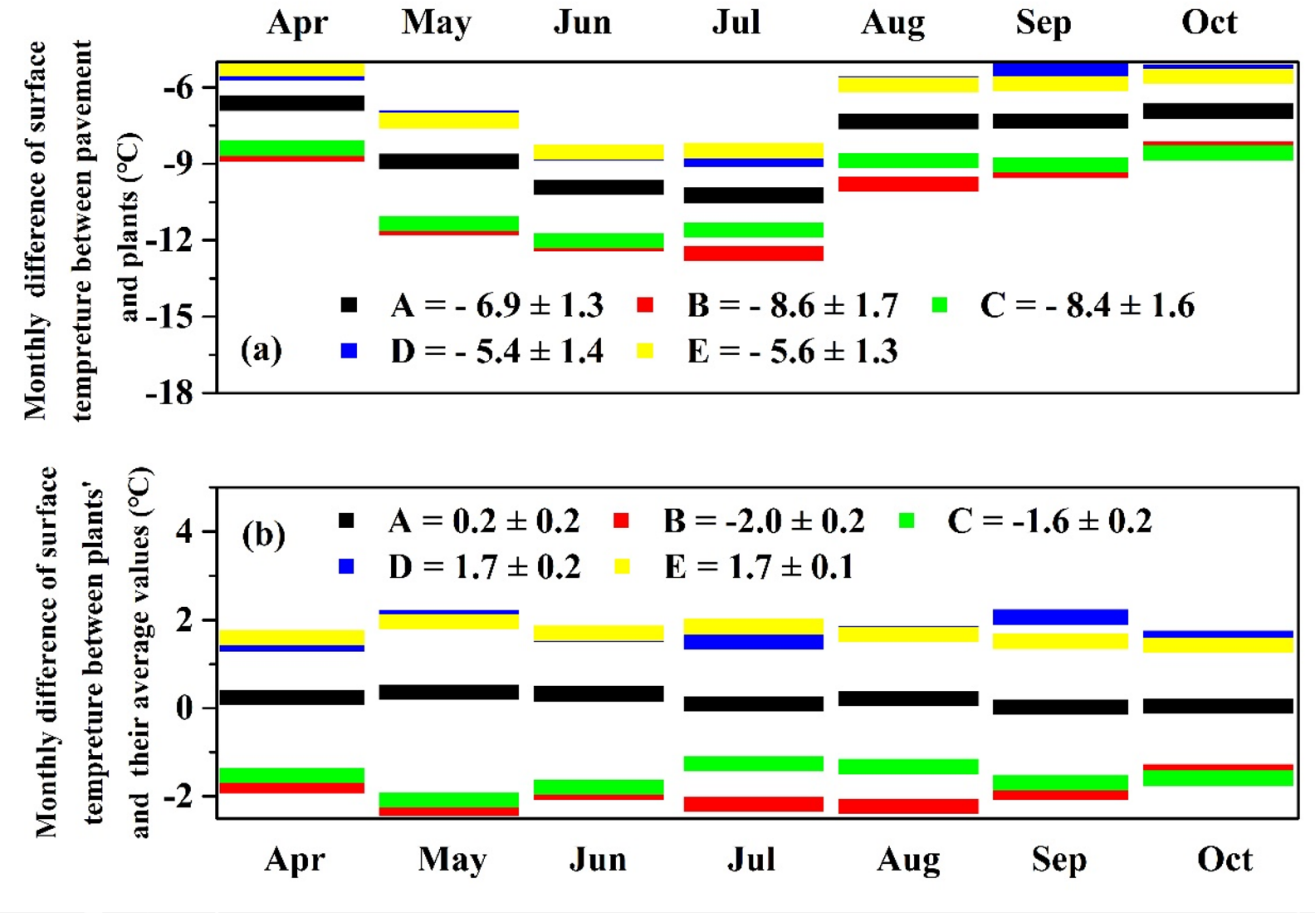
The monthly temperature difference (**a**) between plant surface and pavement, and (**b**) between plant surface and average value. A, B, C, D and E represent *Carmona microphylla* (Lam.) G. Don, *Murraya exotica* L., *Ficus microcarpa ‘Golden Leaves’, Duranta repens* L. and *Hibiscus rosa-sinensis* Linn., respectively.

Figure 4a shows the monthly surface temperature difference between pavement and plants, and the absolute values exhibited noticeable monthly differences. July shows the greatest surface temperature, followed by June, and then May. *Murraya exotica* L. and *Ficus microcarpa 'Golden Leaves'* show the greatest cooling effect on surface temperature from April to October compared to the other three shrubs, with absolute values of 8.4 ± 1.7 °C and 8.6 ± 1.6 °C, respectively.

Figure 4b shows the monthly difference in surface temperature between the shrubs and their average value. Overall, the cooling effect on the surface temperature of *Murraya exotica* L. (− 2.0 ± 0.2 °C) and *Ficus microcarpa ‘Golden Leaves’* (− 1.6 ± 0.2 °C) is significantly higher than the other three shrubs, and, except for in July and August, they exhibited a similar change trend. The values of *Murraya exotica* L. and *Ficus microcarpa ‘Golden Leaves’* in both July and August were 2.2 and 1.3, respectively. The reason for the discrepancy in these months may be related to differences in their growth.

### Monthly temperature difference and its controls

**Figure 5:**
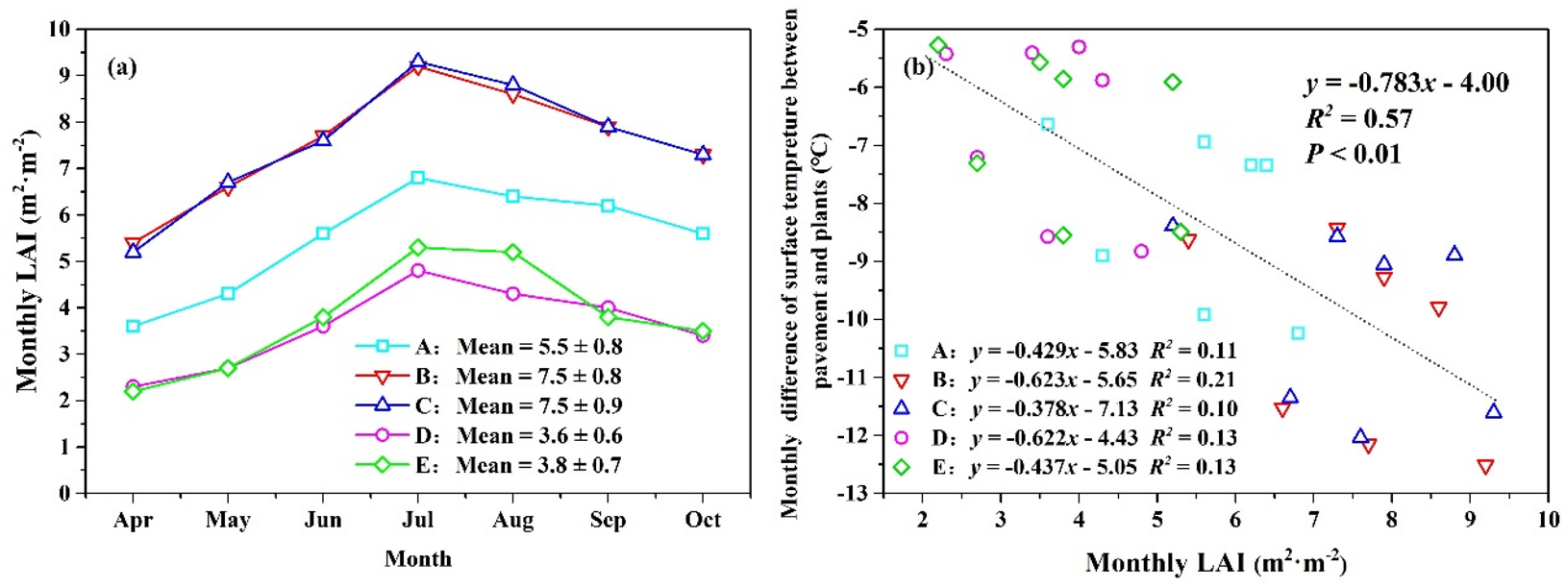
The relationship between monthly LAI (**a**) and monthly temperature difference between plants surface and pavement (**b**). A, B, C, D and E represent *Carmona microphylla* (Lam.) G. Don, *Murraya exotica* L., *Ficus microcarpa ‘Golden Leaves’, Duranta repens* L. and *Hibiscus rosa-sinensis* Linn., respectively.

As shown in Fig. 5a, the LAI of all five shrubs exhibited noticeable seasonal trends. LAI began to increase rapidly in April, peaking in July. It then slowly decreased from July to October. The LAI of different vegetation varied greatly. In particular, the LAI of *Murraya exotica* L. (7.5 ± 0.8 m^2^ m^−2^) and *Ficus microcarpa ‘Golden Leaves’* (7.5 ± 0.9 m^2^ m^−2^) showed larger values than *Carmona microphylla* (Lam.) G. Don (5.5 ± 0.8 m^2^ m^−2^), *Duranta repens* L. (3.6 ± 0.6 m^2^ m^−2^) and *Hibiscus rosa-sinensis* Linn. (3.8 ± 0.7 m^2^ m^−2^) during the entire study period. Figure 5b shows the relationship between the monthly LAI and monthly differences in surface temperature between pavement and plants. They exhibited a strong positive correlation (*R*^2^ = 0.57), meaning that the cooling effect on surface temperature is controlled by vegetation LAI.

**Figure 6:**
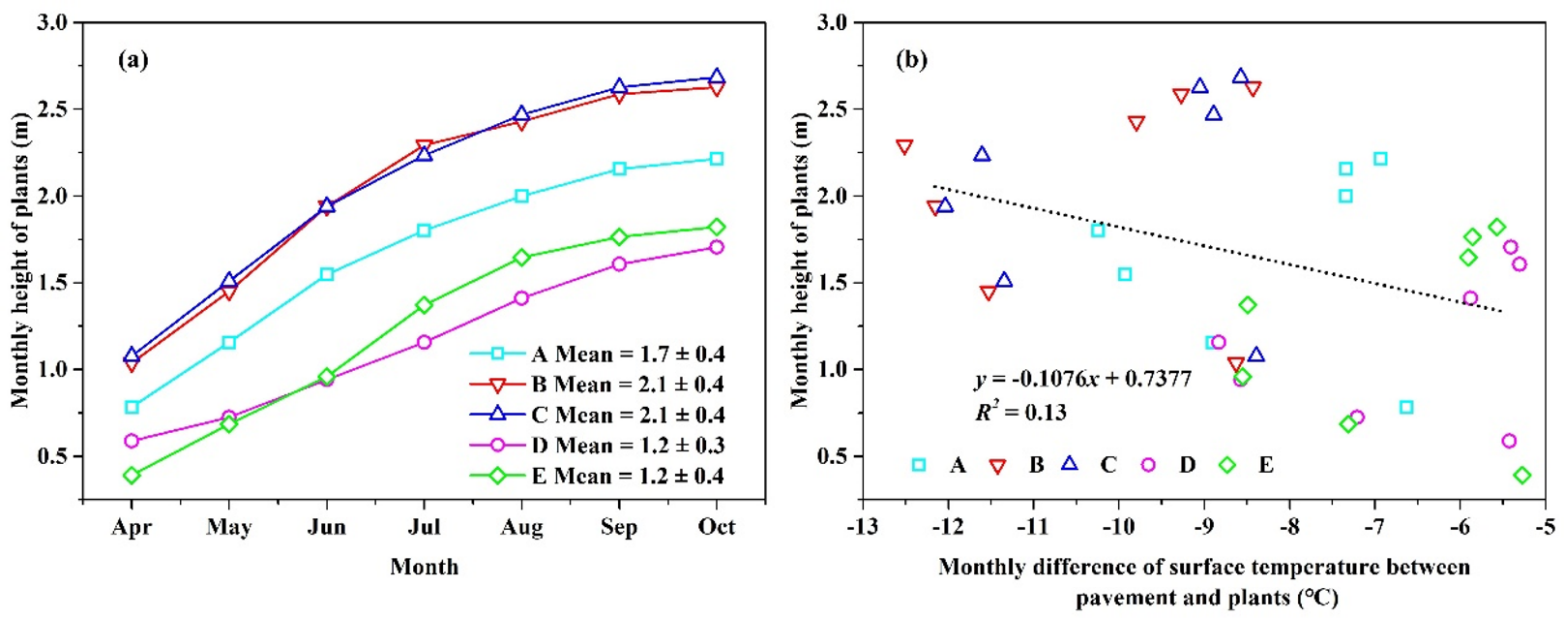
The relationship between the monthly plant height (**a**) and monthly temperature difference between plants surface and pavement (**b**). A, B, C, D and E represent *Carmona microphylla* (Lam.) G. Don, *Murraya exotica* L., *Ficus microcarpa ‘Golden Leaves’, Duranta repens* L. and *Hibiscus rosa-sinensis* Linn., respectively.

Figure 6a shows the difference in changes in height of several shrubs during the study period. *Murraya exotica* L. (2.1 ± 0.4 m) and *Ficus microcarpa ‘Golden Leaves’* (2.1 ± 0.4 m) was taller than *Carmona microphylla* (Lam.) G. Don (1.7 ± 0.4 m), *Duranta repens* L. (1.2 ± 0.3 m) and *Hibiscus rosa-sinensis* Linn. (1.2 ± 0.4 m) during the entire study period. Figure 6b shows the relationship between the monthly height of the plants and monthly difference in surface temperature between pavement and plants. These values also exhibited a positive correlation (*R*^2^ = 0.13), suggesting that plant height influences the cooling effect on surface temperature.

## Discussion

The diurnal maximum surface temperature of shrubs exhibited a similar seasonal variation to that of concrete pavement, with different peak values. The cooling effect of the shrubs on the diurnal maximum surface temperature differed between plant species. The LAI and plant height of different shrubs also exhibited positive influence on the cooling effect.

In this study, the diurnal maximum surface temperature of shrubs (Mean ≤ 40.3 °C) was significantly lower than concrete pavement (Mean = 47.0 ± 4.7 °C) as shown in Fig. 2. The shading effect of the plant canopy contributes to a cooler environment on the surface of the plant^32–34^. The specific heat capacity of leaves is different from that of concrete pavement, which results in their different effects on solar radiation heating^35^. The gaps between the leaves allow direct sunlight to pass through the canopy^36^. The sunlight is blocked by the absorption of the soil surface and the back of the leaf^37,38^. The light reflectivity of the plant canopy is lower than that of concrete pavement, and it also contributes to the higher surface temperature of pavement^39^. More importantly, the transpiration process in shrubs absorbs heat^40,41^ and thus plays a major role in cooling the surface temperature^42,43^.

The LAI is a control factor in the vegetation cooling effect on surface temperature, and these were positively correlated in this study (*R*^2^ = 0.57). It mainly influences the cooling effect of vegetation by affecting transpiration^44,45^. Previous studies have demonstrated that LAI can affect cooling effect through a demand for power of transpiration and transpiration medium^46–49^. For example, Wei et al.^48^ found that LAI is positively related to canopy conductance, which can directly affect transpiration. Bucci et al.^46^ found that LAI is a good indicator of tree density, which is associated with canopy conductance in a neotropical savanna. Li et al.^47^ showed that high LAI signifies a high ratio of transpiration and evapotranspiration. Zhang et al.^49^ showed that increased LAI will increase water demand and transmission power, while increased stomata directly increase the water transport channel.

Taller plants tend to have more biomass and consume more water during the growing period^50,51^. For this reason, plant height can influence the cooling effect on surface temperature (*R*^2^ = 0.13, *p* < 0.01). Many studies have also demonstrated the relationship between height and biomass^51–53^. For example, Ni‐Meister et al.^51^ provided good estimates of wood volume and biomass at the stand level based on the relationship between vegetation height and biomass. Boudreau et al.^52^ found that average stand height and biomass showed a strong correlation, and could be used to estimate regional aboveground forest biomass. Flombaum and Sala^53^ developed a non-destructive and rapid method to estimate biomass and aboveground primary based on the theory that greater plant height results in an increase in biomass per unit area. Plant height also influences the cooling effect because the monitoring point is farther away from the ground, so the light reflected from the ground or soil surface is farther from the monitoring device. Consequently, the measured temperature would be lower. From the perspective of photo thermal theory, thermal radiation gradually decreases as the distance from the hot spot increases^54^.

## Conclusion

In this study, five regionally common shrubs were selected to study the cooling effect and control factors in Guangzhou, southern China. The maximum surface temperature of the five shrubs and pavement was measured and compared using infrared temperature sensors. Results showed that the five shrubs exhibited different cooling effects on surface temperature controlling for the LAI and plant height. The LAI influences transpiration mainly by affecting the water consumption demand of plants and water transport medium, thus influencing the cooling effect. Plant height also affects cooling through biomass and growth water demand. Also, monitoring sensors are farther from ground reflection points, resulting in lower temperatures. This study may have important implications for plant selection in urban green belts designed to reduce the UHI effect.
